# Self-management strategies and multicomponent training to mitigate the effects of the interruption of physical exercise programmes in the pandemic context on functionality, sedentary behaviour, physical capacity, mental health, body composition and quality of life in older adults: a blinded randomized controlled study protocol

**DOI:** 10.1186/s13063-022-06844-2

**Published:** 2022-11-04

**Authors:** Ana Claudia Silva Farche, Gabriela Cassemiliano, Paulo Giusti Rossi, Bianca Ferdin Carnavale, Stefany Lee, Laura Bonome Message, Vinícius Ramon da Silva Santos, Juliana Hotta Ansai, Eduardo Ferriolli, Natália Duarte Pereira, Guillermo Rúben Oviedo, Myriam Guerra-Balic, Maria Giné-Garriga, Marina Araújo Lopes, Anielle Cristhine de Medeiros Takahashi

**Affiliations:** 1 Department of Physical Therapy, Federal University of São Carlos, São Carlos, Brazil; 2 Department of Gerontology, Federal University of São Carlos, São Carlos, Brazil; 3 Faculty of Medicine of Ribeirão Preto, University of São Paulo, Ribeirão Preto, São Carlos, Brazil; 4 School of Psychology, Education and Sport Sciences, Ramon Llull University, Barcelona, Spain; 5 Faculty of Pharmacy and Food Science, University of Barcelona, Barcelona, Spain

**Keywords:** COVID-19, Confinement, Older adults, Multicomponent exercise, Self-management, Randomized controlled trial

## Abstract

**Background:**

Considering the confinement recommended by the World Health Organization due to the pandemic caused by COVID-19, many community physical exercise programmes for older adults have had their activities cancelled. In this context, proposing strategies to recover the possible adverse effects of the confinement period is pertinent. The use of self-management strategies associated with regular physical activity reduces sedentary behaviour and improves physical capacity in older adults. Thus, the purpose of this study was to describe a multicomponent training programme combined with a self-management strategy protocol to mitigate the effects of interruptions in physical exercise programmes on functionality, physical capacity, mental health, body composition and quality of life in older adults.

**Methods:**

This will be a blinded, randomized and controlled clinical trial performed in São Carlos, SP, Brazil. Eighty older adults will be divided into two groups: multicomponent training (Multi) and multicomponent training + self-management strategies (Multi+SM). The intervention will be performed over 16 weeks on three alternate days of every week, with 50-min sessions. The assessment of physical capacity will be performed before the interruption of physical exercise programmes (T0: initial assessment, March 2020), preintervention (T1: immediately after the return of the exercise programme) and postintervention (T2). The assessments of physical activity level, quality of life, mental health, functionality and body composition will be performed at T1 and T2.

**Discussion:**

The results from this MC+SM protocol will allow us to contribute clinical support to evaluate the variables analysed and to guide future public health policies with the aim of minimizing the possible deleterious effects arising from the physical exercise interruption periods caused by epidemics and pandemics.

**Trial registration:**

RBR-10zs97gk. Prospectively registered in Brazilian Registry of Clinical Trials (ReBEC) on 17 June 2021. Registry name: Use of self-management strategies combined with multicomponent training to mitigate the effects of social distancing due to COVID-19 on capacity, physical capacity, mental health and quality of life in older adults - A blind, randomized and controlled clinical trial.

## Administrative information

Note: the numbers in curly brackets in this protocol refer to the SPIRIT checklist item numbers. The order of the items has been modified to group similar items (see http://www.equator-network.org/reporting-guidelines/spirit-2013-statement-defining-standard-protocol-items-for-clinical-trials/).

**Table:** 

Title {1}	Self-management strategies and multicomponent training to mitigate the effects of the interruption of physical exercise programs in the pandemic context on functionality, sedentary behaviour, physical capacity, mental health, body composition and quality of life in older adults: a blinded randomized controlled study protocol
Trial registration {2a and 2b}.	RBR-10zs97gk.
Protocol version {3}	v01 approved on June 30, 2020. Renewal of ethical approval due to COVID delays on October 11, 2021, and June 09, 2022.
Funding {4}	1. FAPESP Process 2020/05471-5 – Role: study design, data collection, analysis, and interpretation; writing the manuscript; 2. CAPES funding cod. 001 – Role: data collection, analysis, and interpretation; writing the manuscript;
Author details {5a}	*Ana Claudia Silva Farche*^*1*^*, Gabriela Cassemiliano*^*1*^*, Paulo Giusti Rossi*^*1*^*, Bianca Ferdin Carnavale*^*1*^*, Stefany Lee*^*1*^*, Laura Bonome Message*^*1*^*, Vinícius Ramon da Silva Santos*^*1*^ *Juliana Hotta Ansai*^*2*^*, Eduardo Ferriolli*^*3*^*, Natália Duarte Pereira*^*1*^*, Guillermo Rúben Oviedo4, Myriam Guerra-Balic4, Maria Giné-Garriga4, Marina Araújo Lopes5 and Anielle Cristhine de Medeiros Takahashi*^*1*^*.* *1. Department of Physical Therapy, Federal University of São Carlos, São Carlos, Brazil* *2. Department of Gerontology, Federal University of São Carlos, São Carlos, Brazil* *3. Faculty of Medicine of Ribeirão Preto, University of São Paulo, Ribeirão Preto, São Carlos, Brazil* *4. School of Psychology, Education and Sport Sciences, Ramon Llull University, Barcelona, Spain* *5. Faculty of Pharmacy and Food Science, University of Barcelona, Barcelona, Spain*
Name and contact information for the trial sponsor {5b}	Ana Claudia Silva Farche, PhD. Department of Physical Therapy, UFSCar – Federal University of São Carlos, R. Washington Luiz, km 235, Zip Code: 13565-905, São Carlos, SP, Brazil. Email: anaclaudiafarche@gmail.com
Role of sponsor {5c}	Each author and coauthor meet all four criteria: 1) Substantial contributions to the conception or design of the work or acquisition, analysis or interpretation of data for the work; 2) Drafting the work or revising it critically for important intellectual content; 3) Final approval of the version to be published; 4) Agreement to be accountable for all aspects of the work in ensuring that questions related to the accuracy or integrity of any part of the work are appropriately investigated and resolved.

## Introduction

### Background and rationale {6a}

The COVID-19 pandemic is a health crisis that has forced the world’s population to live in confinement for several months in an attempt to delay transmission, avoid rapid advances of the disease and minimize the collapse of intensive care units in hospitals [1, 2]. Social restriction recommendations may be a health challenge for the population because confinement can reduce physical activity levels, which has important impacts on muscle, cardiovascular, metabolic, endocrine and nervous systems [3, 4]. Among the populations most affected by these restriction measures, older adults stand out [5, 6].

During ageing, there are numerous physiological changes and a consequent reduction in physical performance as well as in the individual’s ability to maintain homeostasis [6]. Thus, older adults are considered more vulnerable than other confined groups to the onset or worsening of sarcopenia, frailty and cardiometabolic diseases, possibly leading to increased morbidity and mortality in this population [5, 7, 8]. In addition, individuals who present chronic comorbidities also have a greater predisposition to aggravate the disease, which affects most of the population over 60 [2, 5]. Although confinement is an effective measure and must be strictly followed [9], the literature shows negative outcomes for older adults, especially on physical capacity and mental health [9–13]. The lack of social contact is related to a greater risk of depression and anxiety [10], especially during a pandemic situation, as many older adults experience insecurities, increased concern for family members and grieving processes. In addition to psychological symptoms, these measures may lead to a decline in functionality, physical capacity and consequent markers of inactivity, such as cardiovascular, respiratory, neurocognitive and musculoskeletal problems and increased dependence and risk of falls [9–13]. Schrempft et al. (2019) observed that social isolation for older adults is related to a low physical activity level and longer sedentary behaviour. Therefore, the authors suggest that physical inactivity could contribute to the increase in risk factors related to social isolation [13].

In this context, due to the pandemic, many group physical exercise programmes had to be discontinued, and the older population was greatly affected [14]. While confinement of older adults is important to minimize the pandemic situation, measures for immediate recovery after returning to activities and the implementation of preventive strategies for further damage are needed [13]. Thus, regular physical exercise for older adults, especially multicomponent exercise (resistance, aerobic, flexibility and balance), is related to benefits and the preservation of physical, functional and cognitive capacity and a reduction of anxiety, risk of depression and falls. In addition, multicomponent training increases expectations of living independently and improves the quality of life [6, 15]. However, despite a regular practice of moderate-vigorous exercises, the time spent in a sitting or lying position that generates energy expenditure ≤ 1.5 MET can reduce functional independence and increase mortality [16–20].

In addition, self-management (SM) policies have been prominent in the literature. Previous authors have shown SM strategies in which individuals empower themselves with self-care, while professionals instruct patients to take care of their own health, encouraging the development of their autonomy [21]. These SM programmes are described as a primary mechanism for effective chronic disease management [22]. These programmes aim to establish goals for the patients, record their progress and plan their activities. All work is recorded and maintained with the patient, facilitating the personal record of goals, progress and strategies used [16, 23]. Among the themes addressed by SM strategies, there is a focus on reducing sedentary behaviour [16], which may have been aggravated due to social distancing caused by the COVID-19 pandemic [9, 10]. In this context, considering a possible decrease in physical capacity, mental health, physical activity levels and functionality, the association of SM strategies with multicomponent exercises can be a good strategy to mitigate the negative outcomes of social distancing periods for older adults.

### Objectives {7}

The present study protocol aims to describe the design and methods to mitigate the effects of interruptions in physical exercise programmes in the pandemic context on functionality, sedentary behaviour, physical capacity, mental health, body composition and quality of life in older adults using self-management strategies and multicomponent training.

### Trial design {8}

**Fig. 1:**
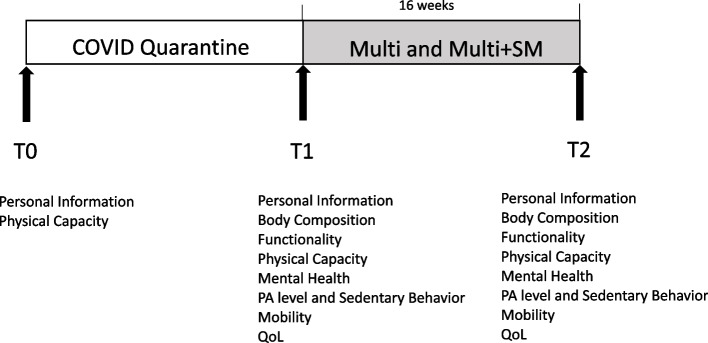
Study timeline. All participants were assessed three times: once before the pandemic confinement (T0: baseline), a second time after PA programmes return to be opened (T1) and a third time after sixteen weeks’ interventions (T2: Multi and Multi+SM)

This study is an exploratory, blinded randomized controlled trial with a parallel group approved by the research ethics committee of the institution (ID: 4.126.247/2020) and registered in a clinical trial registration platform (REBEC ID: RBR-10zs97gk). This protocol follows the standardized guidelines proposed by the Consolidated Standards of Reporting Trials (CONSORT) and SPIRIT. The assessments will be performed at three stages: prephysical exercise programme interruption (T0: initial assessment performed in March 2020), preintervention (T1: immediately after the return of the physical exercise programme) and postintervention (T2: immediately after the intervention protocol) (Fig. 1).

After the physical exercise programme interruption period, the participants will be randomized to one of the two study arms by an independent researcher using Random Allocation Software. According to the sequence generated by randomization, each participant will correspond to a sealed and opaque envelope and then be allocated into one of two groups: (a) the multicomponent training group (Multi) and (b) the multicomponent training + SM group (Multi+SM).

## Methods: participants, interventions and outcomes

### Study setting {9}

This protocol is based on the SITLESS study developed in Europe and is being performed in São Carlos-SP, Brazil. São Carlos is a Brazilian municipality, 254 km from the city of São Paulo, and had a population of 254,484 inhabitants. For this study, all participants of a public physical exercise programme (“Geriatric Revitalization”) were invited, which could reside in any area of the municipality and with a diverse socioeconomic profile.

### Eligibility criteria {10}

The inclusion criteria were as follows: participants aged ≥60 years old who were enrolled in the “Geriatric Revitalization” physical exercise programme coordinated by the institution prior to interruption by the pandemic. The patients will have agreed to participate. The exclusion criteria included indication of cognitive deficit, which will be assessed by Mini-Mental State Examination (MMSE) scores <18 [24], and physical limitations that make it impossible to participate in any of the proposed assessment tests and physical activity programmes.

### Who will take informed consent? {26a}

The study procedures will be presented individually to participants by an investigator, and all questions will be clarified. After that, the informed consent form will be signed voluntarily by both parties.

### Additional consent provisions for the collection and use of participant data and biological specimens {26b}

No biological specimens will be collected as part of this trial.

## Interventions

### Explanation for the choice of comparators {6b}

It is already known in the literature that multicomponent physical exercise has benefits for the physical and mental health of older adults. Thus, to evaluate whether the association of SMS with multicomponent exercises can be a good strategy to mitigate the negative outcomes of the social distance period for this population, the Multi group will be considered a control group for comparing the effect of the inclusion of these techniques with the Multi+SM group.

### Intervention description {11a}

Both groups will start the protocol activities after the return of physical exercise programmes. For this, the participants will be invited to a meeting, and they will receive information about sedentary behaviour. At the end of the meeting, informative material with these topics will be offered.

Multicomponent training will be performed by the Multi and Multi+SM groups. The design of the programme follows the American College of Sports Medicine (ACSM) recommendations and considers the previous physical exercise programme, combining muscular strengthening, flexibility and balance exercises [6]. A 16-week training protocol will be performed on three alternate days every week, with 50-min sessions (Table 1). During the sessions, all participants will use a heart rate monitor (Polar® H10) to monitor HR values and the exercise intensity. The SMS will be performed only by the Multi+SM group. If the intervention programme is effective for the Multi+SM group, the Multi group will also be invited to participate in SM strategies after the end of the study.

**Table 1:** Multicomponent training

Training component	Exercise	Intensity	Progression
Warm-up (10’)	Walk and exercises with music and a ball	Increased progressively at moderate-to-vigorous intensity (according to each individual’s fitness levels)	The progression will be performed by the BORG scale, and activities with moderate intensity will be considered from 4 to 6 points and intense activity as 7–9 points
Balance (10’)	Walk in tandem, and dynamic balance circuits of direction and speed and surface changes	--	Progressive difficulty as tolerated and the progression of these exercises will be performed in relation to the support base, walk in circles and change of direction with increased speed
Muscular strengthening (20’)	Strengthening for the upper limbs, calf exercises; lunges; sit-ups; squats; step up and step down	1–3 sets of 8–12 repetitions, major muscle groups	The exercises load will progress according to the individual's suitability for the initial load
Flexibility/cool down (10’)	Stretching of major muscle groups and breathing exercises	30–60 s of static stretching	--

Adapted from Buto et. al., 2009 [25]

#### Self-management strategies

The self-management techniques to minimize sedentary behaviour will be performed by a single researcher based on the “self-management strategies (SMS)” protocol of the SITLESS multicentre study [16]. Initially, the responsible researcher will perform a remote meeting (familiarization stage) with each participant in the Multi+SM group. The main objectives of this meeting will be to introduce the SM protocol and establish a researcher–participant relationship. Participants and researchers will establish together the functional goals for the participant to be more active every day, especially on days when multicomponent exercises are not developed. These goals will be based on ACSM 2021 guidelines for walking or similar aerobic activities [26] for 150 min a week. Participants will receive an activity diary to record the daily activities and goals achieved.

In addition, the Multi+SM group will also receive a pedometer (Decathlon OnWalk 500 Geonaute, France) for use during the self-management intervention to count data steps and motivate and encourage the participants. To this end, the researcher will provide information material with instructions for use [16].

After the initial contact, the SM protocol will be followed up weekly by telephone contact, with calls lasting up to 20 min. The objective of these contacts is to question the participants regarding the daily activities performed and the number of steps achieved in each activity and to encourage the reduction of sedentary behaviour. The SM protocol will be added concomitantly with the Multi protocol for 16 weeks.

### Criteria for discontinuing or modifying allocated interventions {11b}

There will be no special criteria for discontinuing or modifying allocated interventions.

### Strategies to improve adherence to interventions {11c}

Regular telephone calls will be performed by a physiotherapist with the participants to reinforce the importance of this programme.

### Relevant concomitant care permitted or prohibited during the trial {11d}

Implementing multicomponent physical exercise or self-management strategies will not require alteration to usual care pathways (including the use of any medication), and they will continue for both trial arms.

### Provisions for posttrial care {30}

At the end of the intervention, in case of positive results, both groups will be invited to participate in an SMS programme.

### Outcomes {12}

For this study, the physical activity level and sedentary behaviour are considered the primary outcomes. These variables will be assessed by the energy expenditure (MET/hour), number of steps, cadence, transferences and the spent in each posture preintervention (T1: immediately after the return of the exercise programme) and postintervention (T2).

Secondary outcomes will be verified: (a) body composition in terms of lean mass and relative muscle mass; (b) functionality through an evaluation of cognition, mobility, self-care, interpersonal relationships, living and household activities and participation; (c) physical capacity according to a timed up and go test (time), handgrip strength, MMII strength, balance and 6-min walk test (distance walked); (d) mental health according to depressive symptoms and stress perception; (e) mobility according to a Life-Space Assessment; and (f) quality of life through a structured questionnaire. The outcomes are detailed in Table 2, and the plans for the assessment and collection of outcomes are described in the “Plans for assessment and collection of outcomes {18a}” section.

**Table 2:** Overview of outcomes, outcome measures, instruments and assessment time

Outcomes	Outcome measures	Instrument	Assessment time
Personal information	Age, sex, educational background and medical conditions	Self-reported	T0, T1, T2
Body mass index	Height and weight	Stadiometer/weighing scale	T0, T1, T2
Body composition	Lean mass and relative muscle mass	Dual-energy X-ray absorptiometry	T1, T2
Functionality	Cognition, mobility, self-care, interpersonal relationships, living and household activities and participation	WHODAS 2.0	T1, T2
Physical capacity	Timed Up and Go (time); handgrip strength; MMII strength; balance and 6-min walk test (distance walked)	Timed Up and Go (TUG); manual dynamometer; 30-s sit and stand test; unipodal test; and 6-min walk test	T0, T1, T2
Mental health	Depressive symptoms and stress perception	Geriatric Depression Scale—GSD-15, and Perceived Stress Scale (PSS-10)	T1, T2
Physical activity level and sedentary behaviour	Energy expenditure (MET/h), number of steps, cadence, transferences, and the time spent in sitting and lying postures in the waking period	Accelerometry system activPAL3TM actigraph (PAL Technologies Ltd., Glasgow, UK)	T1, T2
Mobility	Life space	Life-Space Assessment (LSA)	T1, T2
Quality of life	Quality of life	WHOQOL	T1, T2

Adapted from SITLESS protocol [16]. *T0* initial assessment (March/2020), *T1* immediately after the exercise programme return, *T2* postintervention (16 weeks after intervention)

### Participant timeline {13}

The study procedures will be performed at three stages: prephysical exercise programme interruption (T0: initial assessment performed in March 2020), preintervention (T1: immediately after the return of the physical exercise programme) and postintervention (T2: immediately after the intervention protocol) (Fig. 1).

### Sample size {14}

The sample size was calculated by the G*Power software (version 3.1.3, Kiel, Alemanha). Based on data from [22], we estimated that the change from baseline in sitting time adjusted for Accelerometery activPAL3TM actigraph wear time would have a standard deviation of 8.3%. Assuming an 80% follow-up rate, a sample of 60 (30 in each arm) is estimated to provide 80% power to detect a between-group difference in sitting time.

### Recruitment {15}

Participants will be recruited from a public and regular physical activity programme for older adults. All participants will be recruited from the “Geriatric Revitalization” programme. This project began in 2003 and is a partnership between the Federal University of São Carlos (UFSCar) and the São Carlos Educational Foundation (FESC). This project provides periodic physical evaluations three times a year (March, July and December) and systematized physical exercise supervision for 100 older adults. The recruitment will be performed by invitation via a telephone call to all participants with inclusion criteria who will be enrolled by the responsible research physiotherapists.

## Assignment of interventions: allocation

### Sequence generation {16a}

All participants will be randomized and allocated into one of two groups by an independent researcher using the Random Allocation Software (version 2.0, Albuquerque, USA). For this process, there will be no stratification.

### Concealment mechanism {16b}

Initially, each volunteer will be assigned a numeric ID registered in the Random Allocation Software. The randomization sequence will be automatically generated by the software and shared with an independent investigator (TDR). Thereafter, the independent investigator will enter the randomization information into sequentially numbered, opaque and sealed envelopes.

### Implementation {16c}

According to the sequence generated by randomization software, the participants will be allocated into one of the following groups: a group that will follow a multicomponent exercise programme (Multi) and a group that will follow a multicomponent exercise programme plus the self-management (SM) intervention (Multi+SM). The envelopes with randomization information will be opened by the person responsible for the intervention programme (physiotherapist) in the presence of the participant. The professionals responsible for assessing all outcomes and data analysts will have no contact with the envelope.

## Assignment of interventions: blinding

### Who will be blinded {17a}

The professionals responsible for assessing all outcomes and data analysts will be blinded.

### Procedure for unblinding if needed {17b}

The design of this protocol is open label, with only outcome assessors and data analysts being blinded; thus, unblinding will not occur.

## Data collection and management

### Plans for assessment and collection of outcomes {18a}

#### Anamnesis and body composition

When physical exercise programmes return, all participants will participate in an interview structured by a multidisciplinary team. In this anamnesis, the following data will be collected: demographic data (age, years of study, ethnicity, and sex), comorbidities, smoking and alcohol habits prior to COVID-19 diagnosis, and hospitalizations in the last year. The use of medications will be evaluated by a pharmacist.

Body composition will be evaluated by a dual-energy X-ray absorptiometry (DXA) system (Discovery A; Hologic Inc., Bedford, MA, USA) according to the protocol described by Bijlsma et al. [27]. Measurements of lean mass and relative muscle mass [appendicular lean mass/height^2^ (kg/m^2^)] will be obtained [28].

#### Functionality

The functionality assessment will be obtained through the World Health Organization (WHO) Disability Assessment Schedule (WHODAS 2.0). This assessment instrument was developed by the WHO to provide a standardized method of measuring health and disability cross-culturally. The evaluated domains are cognition, mobility, self-care, interpersonal relationships, living and household activities and participation [29]. For this study, the version administered by respondents will be used [29].

#### Physical capacity

Physical capacity will be assessed by the Timed Up and Go (TUG) test [30]; handgrip strength [31]; 30-s sit and stand test [32]; unipodal test [32]; and 6-min walk test [33]. These assessments will be carried out according to the structure presented in Table 2.

#### Mental health

Mental health will be assessed with instruments validated for the Brazilian population considering depressive symptoms and perceived stress. The assessment of depressive symptoms will be performed using the Geriatric Depression Scale (GSD-15). This scale has a sensitivity of 87% and a specificity of 82% for the cut-off score of 4/5 [34]. Volunteers reaching 5 points or more will be considered indicative of depressive symptoms.

The Brazilian version of the “Perceived Stress Scale” (PSS-10) will be used to assess the level of perceived stress in the previous month. The final score ranges from 0 to 40 points, with higher scores denoting a higher degree of stress [35].

#### Physical activity level and sedentary behaviour

To verify the physical activity level and sedentary behaviour, an accelerometry system will be used, using the activPAL3TM actigraph (PAL Technologies Ltd., Glasgow, UK) at assessment moments T1 and T2. The participant will receive guidance and will be instructed to use the monitor continuously for a week. The accelerometer data are transferred via a USB interface to the specific software activPAL version 8.11.6.70, which analyses data and estimates energy expenditure in MET/hour. This variable will be expressed as the average activity per day, excluding days that do not register the full 24 h.

The means of transference from sitting to standing during each day will also be presented, as well as the time spent in each posture. Sedentary behaviour will be defined by the time spent per day in sitting and lying postures in the waking period. In addition, the walking time, number of steps/day and cadence of steps will be calculated.

#### Life-Space Assessment

To verify the confinement adopted by participants, the Life-Space Assessment (LSA) questionnaire [36], translated and validated version for the Brazilian population, will be used [37]. The Brazilian translated and validated version showed adequate reproducibility (internal consistency of 0.92, reliability with ICC of 0.97 (95% CI: 0.95 to 0.98), standard error of measurement: 4.12 points—3%) [37].

### Plans to promote participant retention and complete follow-up {18b}

For participant retention and complete follow-up, the team of physiotherapists and physical educators responsible for the multicomponent training will make motivational conversations during the exercises and reinforce the importance of the study programme and evaluations. As this is a preexisting physical activity programme, the possibility of continuing in the programme after the end of the study is expected to motivate participants. In addition, participants will receive reports with individual feedback on test performance and leaflets to highlight the importance of each activity developed in the study.

### Data management {19}

Data collection will be performed by certified physiotherapists/researchers for all tests performed. Data will be stored on paper (source documents) within each participant’s medical records. The documents will be archived in a specific storage room at the Laboratory of Research in Health of Older Adults (LAPESI) of the Federal University of São Carlos, with restricted access to researchers. Data entry will be performed by two PhD students (G.C. and S.L.) from the research team (double-check). The data will be stored in Microsoft repositories, and the detailed data management plan will be carried out by the DMP tool platform. The data management plan is available from the corresponding author upon request.

### Confidentiality {27}

All sensitive data of participants will be archived in a repository of the Institution following the GDPR. Each participant will receive a numerical code, and personal data will be obliterated in the analysis.

### Plans for collection, laboratory evaluation and storage of biological specimens for genetic or molecular analysis in this trial/future use {33}

As seen in the “Additional consent provisions for the collection and use of participant data and biological specimens {26b}” section above, no biological specimens will be collected.

## Statistical methods

### Statistical methods for primary and secondary outcomes {20a}

Descriptive statistics for all variables will be obtained. Initially, the Shapiro–Wilk test will be used to verify the normality of the data distribution. The paired *t* test or Wilcoxon test will be used to assess changes in physical capacity on confinement, contrasting T0 and T1.

Regarding the intervention, to compare participant characteristics (age, years of study, MMSE and body composition) between groups at T0, the independent *t* test or Mann–Whitney test will be used, respectively, with or without normality of the data. Two-way ANOVA with repeated measures will be applied to assess the effect of interventions between groups and moments (T1 and T2) on functionality, quality of life, physical capacity, mental health, physical activity levels, life space, body composition and sedentary behaviour. The effect size will be calculated by eta partial square (ηp2), and values of 0.02, 0.06 and 0.14 will be considered small, medium and large effects, respectively [38]. The results will be presented in confidence intervals, and the analysis will be conducted according to intention to treat. If any participant presents with a COVID-19 diagnosis, a linear regression will be performed to verify the contribution of these data to the results obtained.

### Interim analyses {21b}

For this protocol, interim analyses are not needed because this is a low-risk intervention and it is not required by the ethics committee and regulatory authorities.

### Methods for additional analyses (e.g. subgroup analyses) {20b}

No additional or subgroup analyses will be performed as part of this trial.

### Methods in the analysis to handle protocol nonadherence and any statistical methods to handle missing data {20c}

The analysis to handle protocol nonadherence will follow the intention-to-treat analysis principles (HOLLIS et al., 1999), with missing data processed by a multiple imputation method. Participants who do not complete the intervention but who perform at least 70% of the sessions will be included in the statistical analysis by intention to treat.

### Plans to provide access to the full protocol, participant-level data and statistical code {31c}

The datasets analysed during the current study and statistical code will be available from the corresponding author upon reasonable request, as will be the full protocol.

## Oversight and monitoring

### Composition of the coordinating centre and trial steering committee {5d}

The coordinating centre of this study is the Laboratory of Research in Health of Older Adults (LAPESI) from the Federal University of São Carlos, Brazil, directed by Prof. Dr. Anielle Takahashi, who will be responsible for recruiting, enrolling and consenting patients; organizing and conducting assessments; and coordinating the intervention programme. LAPESI’s PhD and master team will be responsible for data management and data analysis. These teams meet weekly, and the data review will be performed every six months on reports presented to the ethics committee and to the team of investigators of Ramon Llull University-Barcelona, Spain.

### Composition of the data monitoring committee, its role and the reporting structure {21a}

For this protocol, DMC is not needed because this is a low-risk intervention and it is not required by the ethics committee and regulatory authorities.

### Adverse event reporting and harms {22}

All serious adverse events will be reported by investigators to the ethics committee in the partial reports following the description in the ICF.

### Frequency and plans for auditing trial conduct {23}

The quality and data review for auditing trial conduct will occur every 6 months with a review of all source documents, a review of data entry and a meeting of the responsible teams. In addition, reports of recruitment rate, data quality, protocol deviations and adverse events will be prepared semiannually for submission to the ethics committee. All documents and data analysis will be available for auditing for at least 5 years after the end of the study. The ethics committee will meet to conduct the review throughout the trial period.

### Plans for communicating important protocol amendments to relevant parties (e.g. trial participants and ethical committees) {25}

Regarding protocol amendments, the funding agencies and ethics committee as well as the coordinating centre and the operational teams will be notified. After ethical and regulatory agency approvals, the participants will be reconsented in the informed consent form. In addition, the protocol will be updated in the clinical trial registry, and any deviations will be fully documented using a breach report form.

## Dissemination plans {31a}

The results will be disseminated to the scientific community and relevant groups via publications in scientific journals, presentations at conferences, and reporting of the results in databases, Research Gate, and other social media (Instagram and Twitter).

## Discussion

It is expected that self-management strategies combined with multicomponent training will be effective in minimizing possible negative outcomes resulting from social distancing in the physical capacity, mental health and quality of life of older adults. In addition, we hope that physical intervention combined with SM can reduce participants’ sedentary behaviour. The protocol design was adapted for European success studies and focuses on the main physical domains while considering the local population and individuality of each participant. For the current study, the protocol is easily replicable, as it uses accessible and low-cost materials.

In the long term, it is expected that self-management and health education techniques can help to provide and monitor public physical activity programmes in primary care centres, maximize the benefits and increase the number of older adults cared for involved in self-care at the end of the activity protocol. In addition, we expect that these techniques can be used in the monitoring of older adults who need to be absent from physical training programmes for various reasons, such as in epidemics or pandemics, or even in particular cases, such as absence due to health conditions and transport/locomotion problems, among others. Thus, if self-management strategies are effective, physical activity programmes can be implemented through health education programmes, which would reduce public health expenses and assist in the care of a greater demand.

The authors consider the absence of accelerometry assessment, body composition, functionality and quality of life measurements as a study limitation. These data were not assessed at T0 since they were not part of the quarterly assessment of the physical exercise programme before the pandemic. However, these variables can be compared at T1 and T2 to verify the effect of the intervention, the main objective of this study*.*

## Trial status

Protocol version: v01 approved on June 30, 2020.

Renewal of ethical approval due to COVID delays on October 11, 2021, and June 09, 2022.

Date recruitment began: November 29, 2021.

Approximate date when recruitment will be completed: September 2022.

## Data Availability

The authors affirm that any data required to support the protocol will be supplied upon request.

## Abbreviations

ACSM: American College of Sports Medicine;
CAPES: Coordenação de Aperfeiçoamento de Pessoal de Nível Superior
FAPESP: Fundação de Amparo à Pesquisa do Estado de São Paulo
SM: Self-management
SMS: Self-management strategies
